# A Dual 5**α**-Reductase Inhibitor Dutasteride Caused Reductions in Vascular Density and Area in Benign Prostatic Hyperplasia

**DOI:** 10.1155/2013/863489

**Published:** 2013-01-17

**Authors:** Masayoshi Zaitsu, Akiko Tonooka, Koji Mikami, Mami Hattori, Yuta Takeshima, Toshimasa Uekusa, Takumi Takeuchi

**Affiliations:** ^1^Department of Urology, Kanto Rosai Hospital, 1-1 Kizukisumiyoshi-cho, Nakahara-ku, Kawasaki 211-8510, Japan; ^2^Department of Pathology, Kanto Rosai Hospital, 1-1 Kizukisumiyoshi-cho, Nakahara-ku, Kawasaki 211-8510, Japan

## Abstract

*Objectives*. Dutasteride, a dual 5**α**-reductase inhibitor, is used to treat benign prostatic hyperplasia. Nevertheless, its histopathological effects on the morphometrics of blood vessels and glands are still controversial. The aim here was to assess the histopathological effects of dutasteride in cases of benign prostatic hyperplasia in a retrospective study. *Methods*. Patients with benign prostatic hyperplasia more than 40 cm^3^ in prostatic volume were administered 0.5 mg of dutasteride daily or left untreated prior to receiving a transurethral resection of the prostate. Images of sections stained with hematoxylin/eosin and with anti-CD31 antibody were analyzed. *Results*. In the dutasteride-treated group, the duration of administration was 16.3 ± 8.1 weeks. Artery/arteriole density and vein/venule density in benign prostatic tissue were both lower in the dutasteride-treated group than in the control group. The vein/venule area as a percentage of the whole area was also lower in the dutasteride-treated group, while the artery/arteriole area did not show a significant difference. Glandular/CD31-expressing vessel densities as well as glandular/CD31-expressing vessel areas were comparable between the two groups. *Conclusions*. Dutasteride reduced the artery/arteriole and vein/venule densities and the proportion of vein/venule area in the tissue of patients with benign prostatic hyperplasia.

## 1. Introduction

Benign prostatic hyperplasia (BPH) is a histologic diagnosis that refers to the proliferation of smooth muscle and epithelial cells within the prostatic transition zone, for which dihydrotestosterone (DHT) is the primary androgen responsible in elderly men [1]. As the initial treatment for symptomatic BPH, pharmacotherapy with *α*_1_-adrenergic blockers, 5*α*-reductase inhibitors, antimuscarinic agents, or a combination thereof is often used [2]. Type 1 and type 2 isoenzymes of 5*α*-reductase are present throughout the body [3], and dutasteride, a dual 5*α*-reductase inhibitor, acts competitively and specifically on type 1 and type 2 isoenzymes to inhibit the conversion of testosterone to the more potent DHT [4–6]. In comparison with finasteride, a 5*α*-reductase inhibitor selectively acting on type 2 isoenzyme [7], dutasteride is a 45-fold greater inhibitor of type 1 5*α*-reductase, and a 2.5-fold greater inhibitor of type 2 5*α*-reductase [8–10]. 

Patients with BPH who do not respond suitably to pharmacotherapy often undergo transurethral resection of the prostate (TUR-P), the gold standard for the surgical treatment of BPH, and microwave thermotherapy [11] in order to improve symptoms of bladder outlet obstruction. Several studies assessing clinical and histopathological perioperative effects of dutasteride have been reported [12–14], in which staining of CD31 or CD34 in endothelium was mainly used to detect “microvessels.” Data delineating histopathological effects of long-term dutasteride administration on BPH tissue are still lacking. Therefore, we conducted a retrospective comparative study to analyze histopathological changes in BPH tissue between patients given dutasteride for a relatively long time and controls without preoperative medication.

Blood vessels are composed of aorta, arteries, arterioles, capillaries, venules, veins, and cava. In the prostatic tissue, arteries and arterioles are identifiable with light microscopy as blood vessels with thick walls of obvious muscular media. There are no specific histologic features that accurately distinguish small arteries from larger arterioles. For convenience, arterioles are said to have a diameter of less than 100 *μ*m [15]. On the contrary, veins and venules are identified as blood vessels with thin muscular wall and the latter is also supposed to be less than 100 *μ*m in diameter. Capillaries have neither a muscular media nor an elastic lamella, while a single but complete layer of endothelial cells lies on a basement membrane [15]. Capillaries are generally difficult to distinguish from lymphatic vessels by light-microscopic morphology. Additionally, it was reported that the vascular endothelial markers CD31 and CD34 are occasionally expressed even in lymphatic endothelial cells [16, 17], which may make it more difficult to definitely distinguish capillaries and lymphatic vessels. The definition of microvessels does not appear in textbooks of pathology [15], but seems to be explained as blood vessels of the microcirculatory system, that is, capillaries, arterioles, and venules [18].

In the present study, we assessed parameters concerning arteries and arterioles as well as veins and venules in resected prostate tissue, as they have solid vascular walls and are firmly identified by examining sections stained with hematoxylin and eosin using light microscopy. Additionally, we counted the number of vascular vessels in the tissue by staining endothelial cells with anti-CD31 antibody. Arteries/arterioles and veins/venules are much larger than capillaries, and it is reasonable to hypothesize that they contribute more to the operative blood loss in the perioperative period. The aim of this study was to assess the histopathological effects of dutasteride administration in the tissue of patients with benign prostatic hyperplasia in a retrospective study. Dutasteride reduced artery/arteriole and vein/venule densities and vein/venule area.

## 2. Patients and Methods

### 2.1. Ethics Statement

This study was conducted in accordance with the Helsinki declaration after approval by the Ethics Committee of Kanto Rosai Hospital. The committee approved the use of oral consent documented in the electronic chart for each patient, as the study was retrospective and nonrandomized.

### 2.2. Clinical Design

This is a retrospective study to investigate effects of dutasteride administered before transurethral resection of the prostate in Japanese BPH patients. Twenty-seven BPH patients whose prostate volume determined by transabdominal ultrasonography was more than 40 cm^3^ were enrolled between February 2010 and April 2011. Their serum PSA levels were less than 4.0 ng/mL, otherwise transrectal prostate biopsy had already shown no cancer in the prostate. They had not received LHRH agonists, antiandrogens, estrogens, and 5*α*-reductase inhibitors and had not experienced any type of prostatic surgery or radiation therapy to the pelvis before. Patients with severe cardiovascular disease, liver disease, renal failure, or bleeding tendency were excluded, as they were not suitable for TUR-P. Patients were administered 0.5 mg of dutasteride once daily (GlaxoSmithKline, UK) for some period (*n* = 15), or else they underwent a transurethral resection of the prostate (TUR-P) without dutasteride treatment (*n* = 12). A daily 0.5 mg tablet is the therapeutic dose approved for treating symptomatic BPH in Japan. Age, body height, body weight, body mass index, and duration of administration of dutasteride were recorded preoperatively. The transurethral resection was carried out by three surgeons with irrigation in 3% D-sorbitol (Uromatic S, Baxter, USA). Patients were not transfused with autologous or allogeneic blood of any type in the perioperative period. Operation time for TUR-P, amount of intravenous crystalloid infusion during TUR-P, and weight of resected prostate tissue were recorded as perioperative data. Pre- and postoperative blood hemoglobin (Hb), hematocrit (Hct), and serum sodium (Na) levels were also determined.

### 2.3. Morphometric Analyses of BPH Tissue

As histopathological data, resected prostate tissue was assessed for artery/arteriole density (AD), vein/venule density (VD), and glandular density (GD). Additionally, artery/arteriole area (AA), vein/venule area (VA), and glandular area (GA) were also evaluated as a proportion of the whole area. Resected prostate tissue was fixed in 10% formalin. As a preliminary experiment, some sections were subjected to Elastica Van Gieson staining in order to distinguish between elastic fibers, collagen fibers, and muscle fibers. All sections were stained with hematoxylin and eosin. Additionally, all sections were immunohistochemically stained with anti-CD31 monoclonal antibody (JC70A, DAKO, Denmark) following the manufacturer's recommendations. Then, a section of one chip at the center of the tissue slide as well as an additional four sections of chips around the first section were scanned with a microscopy (BX51, Olympus, Japan) at a magnification of 40. The histology of each section was captured into a Windows computer with a digital camera (DS-Fi1, Nikon Instruments, Japan) using image-analyzing software (NIS-Elements D, Nikon Instruments, Japan). A scale bar of 1000 *μ*m was placed on each image for calibration in the histological analysis. 

Thereafter, images of sections were analyzed with image-analyzing software (Image J, http://rsbweb.nih.gov/ij/) in order to count numbers of arteries/arterioles, veins/venules, and glands in sections stained with hematoxylin and eosin. Outer lines of whole sections were traced with a freehand-selections tool of Image J in order to measure the traced areas. Outlines of the lumens of arteries/arterioles and veins/venules were also traced in a similar manner to measure their intraluminal areas. Intraglandular areas were thresholded and measured also using Image J. The sums of the number of arteries/arterioles or veins/venules in five sections from a patient divided by the sum of the whole areas of five sections were regarded as AD and VD, respectively. GD was similarly calculated. The sums of all artery/arteriole areas or of all vein/venule areas in five sections divided by the sum of the whole areas of five sections were regarded as AA and VA, respectively. GA was similarly calculated. Supposing that vascular lumens are completely round, the diameter (*d*) of a lumen was calculated by the formula; $d=\sqrt{4A/\pi }$, where *A* is the measured area of the lumen. Density and area of CD31-expressing vessels were similarly calculated with sections stained with anti-CD31 antibody. More precisely, outlines of the lumens of CD31-expressing vessels were traced to measure their intraluminal areas. The sums of the number of CD31-expressing vessels in five sections from a patient divided by the sum of the whole areas of five sections were regarded as CD31D. The sums of all areas of CD31-expressing vessels in five sections divided by the sum of the whole areas of five sections were regarded as CD31A.

### 2.4. Statistical Analyses

The statistical analysis was performed using the Mann-Whitney *U* test for intergroup comparisons. Spearman's rank correlation test and the linear regression analysis were performed to analyze correlations between AD/VD and the duration of administration of dutasteride and to draw least squares regression lines. Values were expressed as the mean ± SD. *P* < 0.05 was regarded as statistically significant.

## 3. Results

### 3.1. Clinical Parameters in the Perioperative Period

Characteristics of patients are listed in Table 1. There were no significant differences in age, height, body weight, body mass index, and prostate volume at the first visit between the control and dutasteride-treated groups. In the dutasteride-treated group, the period of dutasteride administration was 16.3 ± 8.1 weeks and reduction in prostate volume was 28.2% ± 30.2%. Perioperative parameters of TUR-P are also listed in Table 1. There were no significant differences in operation time, amount of intravenous crystalloid infusion during TUR-P, weight of resected prostate tissue, and perioperative changes in Hb, Hct, and serum Na. 

### 3.2. Morphometric Effects of Dutasteride Administration on BPH Tissue

**Figure 1:**
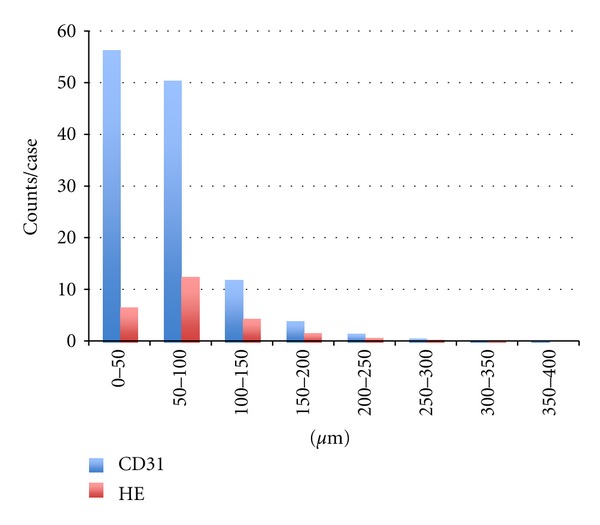
Histogram of calculated diameters of vascular vessels. Horizontal line: diameters of lumens, blue bars: CD31-expressing vessels, and red bars: artery/arteriole and vein/venule. The difference in the number of vessels detected either with hematoxylin/eosin staining or with CD31 staining was more in vessels whose diameters were less than 100 *μ*m compared with larger vessels.

**Figure 2:**
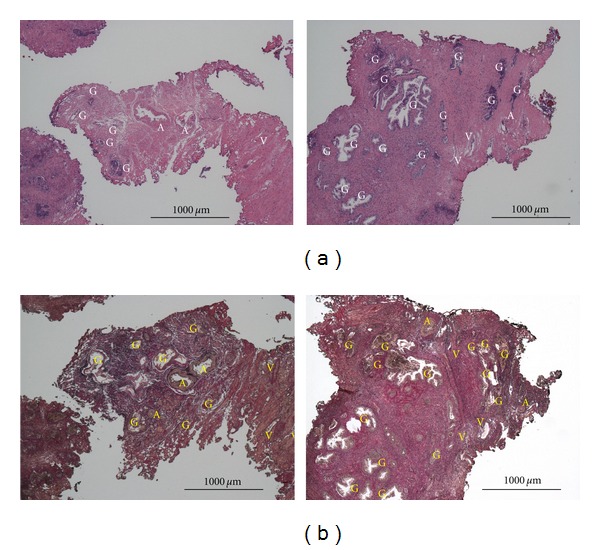
Upper panels: a representative light micrograph of a resected prostatic chip. Upper panels: hematoxylin and eosin staining; Lower panels: Elastica Van Gieson staining. A: artery/arteriole, V: vein/venule, G: gland, and bar = 1000 *μ*m.

Prostatic cancer was not detected in any of the resected prostatic samples. The numbers of vascular vessels detected in all 27 cases either with hematoxylin/eosin staining or with CD31 staining were 724 and 3390 in total, that is, 26.8 and 125.6/case, respectively. The difference in the number of vessels detected either with hematoxylin/eosin staining or with CD31 staining was more in vessels whose diameters were less than 100 *μ*m compared with larger vessels, as shown in Figure 1.  Figure 2 shows the typical histology of BPH with hematoxylin and eosin staining and with Elastica Van Gieson staining. Arteries/arterioles and veins/venules were able to be distinguished by the thick muscular media. As the identification of arteries/arterioles and veins/venules was similar between Elastica Van Gieson staining and staining with hematoxylin and eosin, the histological data for arteries/arterioles and veins/venules were basically obtained using sections stained with hematoxylin and eosin. 

**Figure 3:**
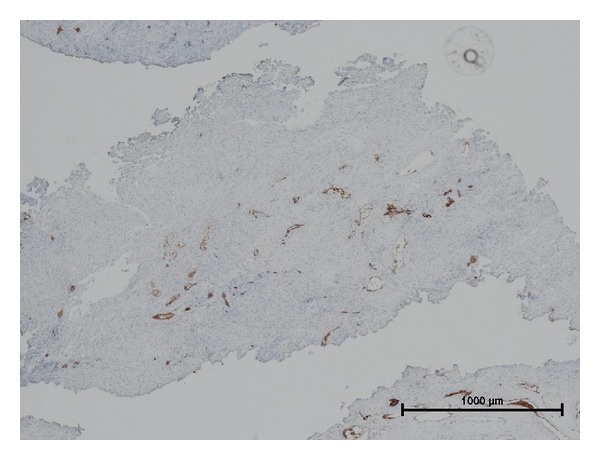
A representative image of a resected prostatic chip stained with the anti-CD31 monoclonal antibody, bar = 1000 *μ*m.

With hematoxylin/eosin staining, the numbers of arteries/arterioles and veins/venules were 182 and 542 in all 27 cases, that is, 6.7 and 20.1/case, respectively, while vessels without media such as capillaries were not counted. Arteries/arterioles, veins/venules, and capillaries were essentially indistinguishable with CD31 staining.  Figure 3 shows the typical histology of BPH with CD31 staining. Histological data for the resected BPH tissues are listed in Table 2. AD, VD, and VA were significantly lower in the dutasteride-treated group than the control group. AA tended to be lower in the dutasteride-treated group. GD and GA were comparable between the groups. CD31D and CD31A were also similar between the two groups.

**Figure 4:**
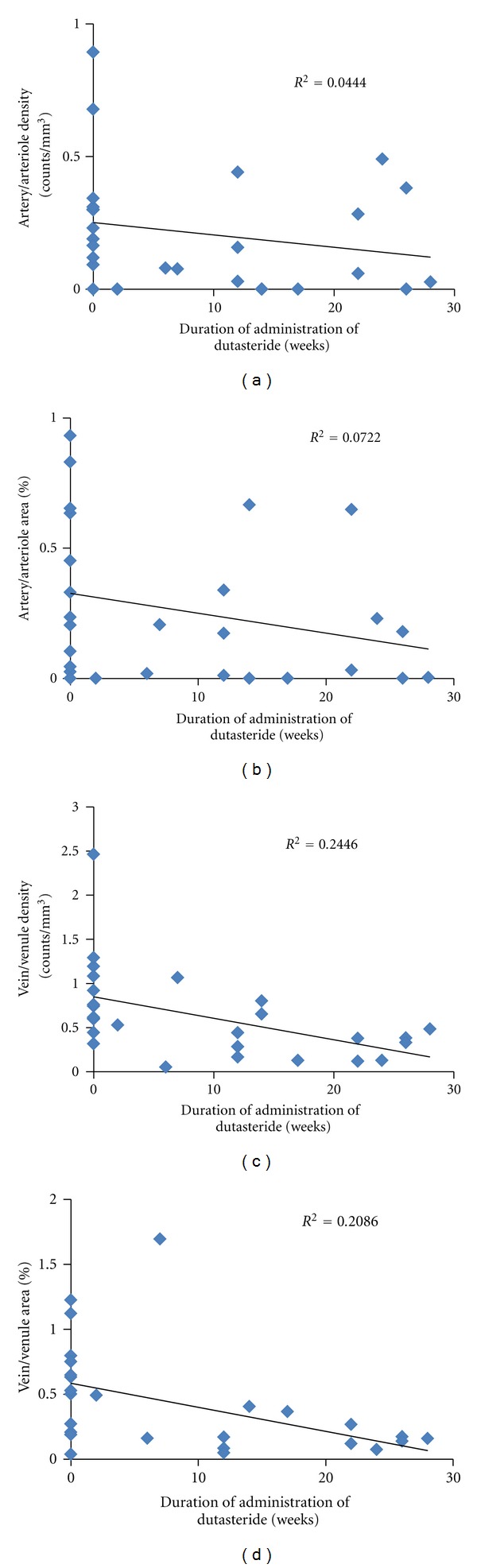
(a) The correlation between AD and the duration of administration of dutasteride. AD: artery/arteriole density, *P* = 0.099. (b) The correlation between AA and the duration of administration of dutasteride. AA: artery/arteriole area as a proportion of the whole area. *P* = 0.089. (c) The correlation between VD and the duration of administration of dutasteride. VD: vein/venule density, *P* = 0.002. (d) The correlation between VA and the duration of administration of dutasteride. VA: vein/venule area as a proportion of the whole area. *P* = 0.003. Data for BPH tissues in the control group are plotted as duration 0. *P* values are for the Spearman's rank correlation test. A least squares regression line was drawn for each panel.

As shown in Figure 4 (lower panels), VD and VA were negatively correlated with the duration of administration of dutasteride (*P* = 0.002 and 0.003, resp.). Least squares regression lines were drawn. Neither AD nor AA showed a significant correlation with the duration of dutasteride treatment (*P* = 0.099 and 0.089, resp., Figure 3 (upper panels)). CD31D and CD31A were not correlated with the duration of administration either (data not shown).

## 4. Discussion

Blood vessels allow haematopoietic cells to eliminate the organism for immune surveillance, supply oxygen/nutrients, and dispose of waste leading to beneficial effects for tissue growth and regeneration. Angioblasts differentiate into endothelial cells and then assemble to form a vascular vessel (vasculogenesis) followed by subsequent sprouting expansion of the vascular network (angiogenesis) [19]. Human and rat prostate endothelial cells play an important role in androgen responses in the prostate, as they express functional androgen receptor and are androgen sensitive [19, 20].

Hahn et al. [12] showed that the perioperative administration of dutasteride had no effects on microvessel density (MVD) in prostatic tissue of BPH patients given pre-operative dutasteride for 2 or 4 weeks compared with placebo controls in a prospective, randomized, and multicenter study, where MVD was counted by the number of microvessels immunostained with anti-CD34 antibody within an area of 0.754 mm^2^ of a resected chip. Accordingly, it follows that there are approximately 70 microvessels per mm^2^ in their control specimens, while the sum of the arteries/arterioles and veins/venules in the control specimens in our study was only 1.22 per mm^2^. Thus, most of the microvessels counted by Hahn et al. are regarded as capillaries and possibly CD34-expressing lymphatic vessels. Ku et al. [14] also showed that dutasteride had no effect on prostatic MVD in BPH patients given pre-operative dutasteride for 2 to 4 weeks compared with controls given no drug, where MVD was calculated as a mean of counts of CD34-positive microvessels in 10 consecutive high-power fields. The CD31D, density of CD31-positive vessels, is much lower in the present study compared with the densities of microvessels in other studies. The distribution of CD31-positive vessels is not even throughout the resected prostatic chips with some portion containing few vessels. In the present study, all CD31-positive vessels in five chips were counted, then the CD31D was calculated by dividing the number of the vessels by the whole area of those chips. Thus, the calculated densities were not very high.

Concerning preoperative finasteride administration and MVD in resected prostatic tissue, Donohue et al. reported that inhibition of expression of vascular endothelial growth factor and reduction of MVD were observed after two weeks administration of 5 mg daily of finasteride, where MVD was assessed by counting CD31-positive microvessels in 10 nonoverlapping, consecutive high-power fields [21]. Furthermore, Lekas et al. [22] reported in a prospective, randomized study that finasteride administration for 25.3 weeks on average to BPH patients resulted in the suppression of MVD as well as vascular endothelial growth factor and hypoxia-inducible factor-1*α* expression in a time-dependent manner, where MVD was assessed by counting CD34-positive microvessels in the three most vascularized high-power fields.

The vessels with media identified with hematoxylin/eosin staining in the present study seem mainly to be arterioles and venules of relatively smaller diameter, as the peak of the distribution of calculated diameters of those vessels was 50–100 *μ*m. The duration of dutasteride administration of 16.3 weeks in the present study was relatively long compared with that in previous studies, up to 4 weeks. This may be why dutasteride administration before TUR-P in this study caused reductions of AD, VD, and VA in the prostatic tissue partly in a time-dependent manner. Otherwise, the counting of numerous capillaries in other reports might have concealed more important influences of dutasteride on larger blood vessels. In other words, although larger blood vessels tend to be influenced by dutasteride more than capillaries, the much more numerousness of the latter contributes to the computed values of the vessel densities and gets changes of the larger vessels indiscernible. Actually, androgens are reported to limit the number of new vessels developed while they contribute to the presence of larger vessels [23] and androgen deprivation therapy induces changes in relatively large vessels [24]. Measurements of luminal areas of blood vessels in the prostate were not made in the preceding reports describing effects of 5*α*-reductase inhibitors, which can be a useful tool for evaluating effects of drugs on blood vessels. As they showed clearer effects of dutasteride including time dependency, veins/venules might be more susceptible to dutasteride than arteries/arterioles. CD31D and CD31A were comparable in the control and dutasteride-treated groups. This may be because the numbers of capillaries in the BPH tissues outweigh those of larger vessels with media and capillaries may not be influenced by dutasteride. Additionally, CD31 might have picked up lymphatic vessels as well. Then, it may be worth evaluating larger vascular vessels with media by morphologically and morphometrically investigating specimens under direct vision and not only relying on staining with endothelial markers such as CD31 and CD34.

In the rat prostate, levels of angiogenetic factors such as hypoxia-inducible factor-1*α* and vascular endothelial growth factor (VEGF) were lower in the dutasteride-treated groups than in the control group [14]. In human BPH tissues, VEGF expression was significantly associated with androgen receptor and type 2 5*α*-reductase expression, but not with type 1 5*α*-reductase expression [25]. These result could support the present finding of decreased vascularity in BPH tissues with dutasteride administration possibly through the suppression of androgen signaling pathways.

Andriole et al. reported that prostate cancer patients administered 10 mg of dutasteride daily for 1 week followed by 5 mg daily for more than 45 days in total showed a decrease in MVD by 45% in cancer tissue in addition to a trend toward increased apoptosis of cancer cells when compared with controls without dutasteride administration [26]. In this paper, the dose of dutasteride was much higher than that used for BPH patients, and so the significant decrease in MVD in prostatic tissue might have been demonstrated after a shorter period of dutasteride administration than in the present paper. Another possibility is that the interstitial microvessels in malignant prostatic tissue differ from those in benign tissue in quality. Andriole et al. also reported in a more recent article that the administration of 0.5 mg of dutasteride daily over a 4-year study period (REDUCE study) reduced the risk of incident prostate cancer and acute urinary retention by 22.8% and 77.3%, respectively, compared with the control [27].

As the administration of finasteride for as little as two weeks reduced prostatic MVD [15], the actions of finasteride on prostatic microvessels may be faster and more profound than those of dutasteride. Additional inhibition of the type 1 5*α*-reductase by a dual 5*α*-reductase inhibitor may antagonize the microvessel suppressive activity of type 2-specific 5*α*-reductase inhibitors.

In benign prostatic tissue, we did not detect differences in glandular density and the proportion of intraglandular area regardless of dutasteride administration. Such morphometrics seem rarely reported, probably because glands in benign prostatic tissue have complicated folds. We could calculate values by thresholding intraglandular spaces with the thresholding function of Image J. In the report by Andriole et al. described above [17], dutasteride caused a decrease of 18% in the width of benign prostatic epithelial cells in patients with prostate cancer. Considering that densities and areas of arteries/arterioles and veins/venules, but not glands in benign prostatic tissue decreased with dutasteride as reported in this study, the main target of dutasteride in reducing BPH size could be the interstitium including blood vessels, not glands.

Perioperative 4–6 weeks' administration of dutasteride had no effect in reducing blood loss during or after TUR-P [12, 13], while two patients in the control group in the latter article required a postoperative blood transfusion compared to no patient in the dutasteride-treated group. Similarly in the present study, blood loss was comparable between the two groups as estimated from the perioperative changes in blood hemoglobin and hematocrit. Thus, the reduction in the density and area of blood vessels in BPH did not directly lead to decreased blood loss in the perioperative period. In conclusion, dutasteride administration for 16.3 ± 8.1 weeks before TUR-P caused reductions of AD, VD, and VA in BPH tissue partly in a time-dependent manner. Blood loss estimated by the perioperative changes in blood Hb and Hct was comparable between the control and dutasteride-treated groups.

## Figures and Tables

**Table 1:** Characteristics of Patients and TUR-P.

	Control group	Dutasteride group	*P* value
Number	12	15	
Age (years)	68 ± 8	71 ± 5	0.1294
Height (cm)	166.6 ± 5.6	166.4 ± 5.5	0.9609
Body weight (kg)	62.2 ± 7.7	64.1 ± 12.0	0.7508
Body mass index	22.3 ± 1.9	23.1 ± 3.7	0.4792
Prostate volume at first visit (cm^3^)	59.4 ± 14.9	70.5 ± 21.9	0.1021
Duration of dutasteride administration (weeks)	—	16.3 ± 8.1	
Reduction in prostate volume (%)	—	28.2 ± 30.2	
Operation time (min)	83 ± 23	80 ± 21	0.9027
Operative intravenous crystalloid infusion (mL)	894 ± 312	702 ± 246	0.1011
Weight of resected prostate tissue (g)	24.4 ± 14.7	18.1 ± 10.4	0.3528
Hb (g/dL)			
Before TUR-P	14.3 ± 1.3	14.0 ± 1.2	0.6080
After TUR-P	12.9 ± 1.3	13.1 ± 1.5	0.7320
Changes in Hb (g/dl)	1.4 ± 0.9	0.9 ± 0.8	0.3167
Hct (%)			
Before TUR-P	42.0 ± 5.8	42.3 ± 3.6	0.9222
After TUR-P	39.0 ± 3.6	39.2 ± 4.3	0.6429
Changes in Hct (%)	2.9 ± 4.5	3.1 ± 2.1	0.9611
Na (nmol/L)			
Before TUR-P	140 ± 2	139 ± 2	0.9603
After TUR-P	139 ± 4	140 ± 3	0.7125
Changes in Na (nmol/L)	1 ± 4	−1 ± 2	0.3894

Hb: blood hemoglobin, Hct: hematocrit, and Na: serum sodium. Values are exBeforessed as the mean ± SD. *P* values are from the Mann-Whitney *U* test.

**Table 2:** Histological data for the resected BPH tissue.

	Control group	Dutasteride group	*P* value
Artery/arteriole density (counts/mm^2^)	0.30 ± 0.25	0.13 ± 0.18	0.0328
Vein/venule density (counts/mm^2^)	0.92 ± 0.57	0.40 ± 0.28	0.0025
CD31-positive vessel density (counts/mm^2^)	3.65 ± 1.46	4.05 ± 1.66	0.5914
Glandular density (counts/mm^2^)	4.21 ± 1.96	4.54 ± 3.12	0.9611
Artery/arteriole area relative to the whole area (%)	0.37 ± 0.33	0.18 ± 0.23	0.0563
Vein/venule area relative to the whole area (%)	0.58 ± 0.37	0.29 ± 0.41	0.0084
Glandular area relative to the whole area (%)	7.94 ± 4.17	7.82 ± 6.19	0.6256
CD31-positive vessel area relative to the whole area (%)	1.85 ± 0.74	1.99 ± 1.08	0.8836

Values are expressed as the mean ± SD. *P* values are from the Mann-Whitney *U* test.
